# MRI-Derived Biomarkers and Radiomic Signatures for Early, Dose-Dependent Evaluation of Prostate Cancer Radiotherapy: An Exploratory Study

**DOI:** 10.3390/jimaging12050213

**Published:** 2026-05-17

**Authors:** Eleni Bekou, Admir Mulita, Ioannis M. Koukourakis, Nikolaos Courcoutsakis, Athanasia Kotini, Evlampia Psatha, Georgios Tsakaldimis, Ioannis Seimenis, Michael I. Koukourakis, Efstratios Karavasilis

**Affiliations:** 1 Medical Physics Laboratory, School of Medicine, Democritus University of Thrace, 68100 Alexandroupolis, Greece; akotini@med.duth.gr (A.K.); ekaravas@med.duth.gr (E.K.); 2 Department of Radiotherapy/Oncology, University Hospital of Alexandroupolis, Democritus University of Thrace, 68100 Alexandroupolis, Greece; mkoukour@med.duth.gr; 3 Department of Clinical Radiation Oncology, School of Medicine, Attikon Hospital, National and Kapodistrian University of Athens, 12462 Athens, Greece; ikoukourakis@med.uoa.gr; 4 Department of Radiology, School of Medicine, Democritus University of Thrace, 68100 Alexandroupolis, Greece; ncourcou@med.duth.gr (N.C.); epsatha@med.duth.gr (E.P.); 5 Department of Urology, Democritus University of Thrace, 68100 Alexandroupolis, Greece; gtsakald@med.duth.gr; 6 Medical Physics Laboratory, School of Medicine, National and Kapodistrian University of Athens, 11527 Athens, Greece; iseimen@med.uoa.gr

**Keywords:** magnetic resonance imaging, MRI biomarkers, radiomics, dose-dependent imaging, treatment response evaluation, radiotherapy, prostate cancer

## Abstract

This study provides an accurate assessment of radiotherapy-induced tissue changes in prostate cancer when relying solely on serum prostate-specific antigen kinetics. The current study aims to explore the role of quantitative magnetic resonance imaging and radiomic analyses. In this exploratory prospective study, 22 patients with histologically confirmed prostate cancer underwent multiparametric magnetic resonance imaging at three time points: pre-treatment, mid-treatment, and two months post-radiotherapy. Quantitative imaging analysis included total prostate volume, T2, apparent diffusion coefficient—ADC, and T2* mapping, alongside T2-weighted and diffusion-weighted radiomic feature extraction. Longitudinal changes and dose correlations were analyzed using repeated-measures ANOVA and linear mixed-effects models. Prostate volume increased from 44.22 ± 21.26 cm^3^ at baseline to 51.11 ± 22.36 cm^3^ mid-treatment (*p* < 0.001) and decreased to 37.98 ± 15.5626 cm^3^ post-treatment (*p* = 0.034), indicative of temporary radiation-induced glandular edema. T2 relaxation times decreased from 106.00 ± 23.74 ms to 93.33 ± 9.50 ms after therapy (*p* = 0.023), with androgen deprivation therapy influencing overall values (partial η^2^ = 0.228, *p* = 0.028), while ADC and T2* remained largely stable (*p* > 0.05). Radiomic features, particularly from DWI, exhibited subtle time- and dose-dependent variations. Radiation dose was significantly associated with volume and T2, but not with ADC or T2*. These findings suggest that quantitative MRI biomarkers combined with radiomic analysis may provide objective, non-invasive measures of early prostate cancer radiotherapy-induced changes. These imaging-derived metrics may capture early treatment-related tissue alterations and could provide exploratory signals for early treatment evaluation in prostate cancer, although their relationship with biochemical markers requires further validation.

## 1. Introduction

Traditionally, therapeutic response evaluation to radiotherapy has relied on clinical and biochemical markers such as prostate-specific antigen (PSA) [1]. Nevertheless, this biomarker demonstrates limited accuracy, exhibiting false-positive elevations that are not associated with tumor recurrence or residual disease, particularly in early disease stages or focal therapies [2,3].

The relatively high incidence (approximately 25%) of local biochemical recurrence or metastatic disease progression among patients with prostate cancer (PCa) undergoing radiotherapy requires additional complementary diagnostic modalities to improve clinical management and prognostic stratification in this population [4,5].

In the last decade, multiparametric magnetic resonance imaging (mpMRI) has been increasingly endorsed for integration into the assessment of active surveillance for men with PCa following radiotherapy (RT). Initially, in 2014, the National Institute for Health and Care Excellence (NICE) supported the use of mpMRI as a key component of risk-adapted management strategies [6]. Subsequently, in 2021, an international expert consensus panel comprising representatives from the European Society of Urogenital Radiology (ESUR), the European Society of Urogenital Imaging (ESUI), and the Prostate Imaging Reporting and Data System (PI-RADS) Steering Committee established a standardized reporting system specifically designed for post-treatment assessment: Prostate Imaging for Recurrence Reporting (PI-RR) [7,8,9].

The most recent PI-RADS v2.1 guidelines, recognizing the limitations of mpMRI—including observer dependence, lengthy scan times, and potential contrast-related risks—recommend the use of biparametric MRI (bpMRI), which includes only T2-weighted and diffusion-weighted imaging, for post-treatment assessment of prostate cancer [10].

The incorporation of quantitative metrics, such as volume, T2 relaxation time, apparent diffusion coefficient (ADC) and T2*, further improves objectivity, reproducibility, and diagnostic accuracy. Moreover, the domain of radiomic analysis allows for the extraction of a wide range of objective features that reflect tissue structure and RT-related modifications [11,12,13].

This study aimed to evaluate the longitudinal RT-induced changes in patients with PCa using advanced MRI techniques, performing a comparative analysis of MRI-derived imaging biomarkers at three time points: before treatment initiation (t0), at the midpoint of radiotherapy (t1), and two months after treatment completion (t2).

The specific objectives of the study include:

- Investigating radiotherapy-related changes in quantitative MRI biomarkers such as total prostate volume, ADC, T2, and T2*,
- Extracting and analyzing variations in radiomic features from MRI data acquired from radiotherapy,
- Assessing the relationship between the MRI biomarkers and the delivered radiation dose.

## 2. Materials and Methods

This prospective exploratory study was held from September 2023 to November 2025, including 22 patients with histologically confirmed PCa who were referred to RT at the Radiotherapy Department of the University Hospital of Alexandroupolis. The study was conducted in collaboration with the Magnetic Resonance Imaging Department at the University Hospital of Alexandroupolis. All participants were examined using a 1.5 T Achieva MR scanner at three different time points:

- t0: Before radiotherapy (baseline),
- t1: Middle of radiotherapy sessions (mid-treatment),
- t2: Two months after completion of radiotherapy (follow-up).

The Ethics and Research Committee of the University Hospital of Alexandroupolis approved the study (No. ES2 12-01-2023). All patients provided written informed consent, authorizing the anonymous use of their clinical and laboratory data for research and publication.

### 2.1. MRI Acquisition and Post-Processing

All patients were scanned with a 1.5T Achieva MRI scanner (Philips Healthcare, Best, The Netherlands). The multiparametric MRI (mp-MRI) protocol consisted of axial, sagittal and coronal high-resolution T2-weighted turbo spin echo sequences, axial plane diffusion weighted imaging (DWI) sequence with diffusion-encoding b-values ranging from 0 to 1200 s/mm^2^, an axial multi-echo TSE (T2_calc) sequence, an axial multi-echo fast field echo (mFFE) sequence with different echo times, and an axial T1-weighted T1 W_mDIXON sequence.

Quantitative T2 maps were generated from the multi-echo turbo spin echo (TSE) sequence (FOV: 144 × 144 × 33 mm^3^, slice thickness: 3 mm, voxel spacing: 1.18 × 1.18 × 3.3 mm^3^, TR: 3242.1 ms) with echo times (TE) ranging from 0 to 160 ms in 20 ms intervals, using voxel-wise mono-exponential least-squares fitting of the signal decay. Apparent diffusion coefficient (ADC) maps were derived from the diffusion-weighted sequence (FOV: 112 × 112 × 25 mm^3^, slice thickness: 3 mm, voxel spacing: 1.33 × 1.33 × 3 mm^3^, TR/TE: 4283.6/78.2 ms), acquiring the following b-values: 0, 50, 80, 100, 150, 200, 500, 800, and 1200 s/mm^2^ using a mono-exponential diffusion model with least-squares fitting of the logarithm of signal intensity versus b-values. T2* maps were obtained from the mFFE sequence (FOV: 80 × 80 × 20 mm^3^, slice thickness:3 mm, voxel spacing: 2.25 × 2.25 × 4 mm^3^, TR: 1435.2 ms) using R2 starmapping code implemented in MATLAB (v. R2022b) based on mono-exponential least-squares fitting across eight echo times (TE): 0.6, 13.8, 23, 32.2, 41.4, 50.6, 59.8, and 69 ms [14,15].

### 2.2. External Beam Radiotherapy Protocol for Prostate Cancer

Simultaneous integrated volumetric modulated arc therapy (SIB-VMAT) delivered with image-guided radiotherapy (IGRT) permitted dose escalation to the primary target (prostate) while minimizing radiation exposure to adjacent neighboring organs at risk (OARs), such as the rectum and urinary bladder. SIB-VMAT with the IGRT technique was applied with a 6-MV Elekta Infinity Linear Accelerator (Elekta, Stockholm, Sweden) equipped with an Agility head (Elekta). The treatment plans were created using Monaco Treatment Planning System (TPS) version 6.1.4.0 (Elekta CMS, Maryland Heights, MO, USA) [16].

Our department followed two distinct therapeutic regimens: the ultra-hypofractionated accelerated regimen (ultra-hypoAR) and the hypofractionated accelerated regimen (hypoAR). Further radiotherapy details are included in Table 1.

The dose was prescribed to the International Commission on Radiation Units & Measurements (ICRU) reference point for PCa [17,18,19]. The plans were optimized to maximize the dose of the planning treatment volume (PTV) while minimizing the dose of the surrounding normal tissues.

Image-guided RT using cone-beam computed tomography (CBCT) before each radiation treatment was performed with the Elekta platform Synergy kV CBCT (XVI) (Elekta AB, Stockholm, Sweden) to assess and adjust the patients’ positions.

### 2.3. Androgen Deprivation Details

High-risk patients—defined as having one or more of the following features: clinical stage T3, positive lymph nodes (N+), PSA levels > 15 ng/mL, or a high Gleason score (GS ≥ 7, particularly 4 + 3)—underwent androgen deprivation therapy (ADT) of luteinizing hormone–releasing hormone (LH-RH) agonists with or without combination with bicalutamide. Therapy was initiated three months prior to the start of radiotherapy and continued for a total duration of 12 months.

### 2.4. Monitoring and Evaluation Protocol

Patients underwent regular biochemical monitoring of PSA every six months to enable early detection of potential local or systemic disease recurrence.

### 2.5. Segmentation

A radiologist experienced with prostate imaging manually delineated the whole prostate gland as well as suspicious lesions, using ITK-SNAP v.3.6.0. [20]. In order to avoid mask misregistration problems, different masks were created for each imaging technique. The created masks were applied in parametric maps (T2 map, T2* map, ADC map) to evaluate the correspondence parameters and radiomics features. It should be noted that quantitative MRI biomarkers were derived at the prostate level, whereas radiomic features were extracted at the lesion level. Therefore, these two types of analyses were performed independently and are not directly comparable.

### 2.6. Radiomics Analysis

Firstly, bias correction was applied on T2-weighted (T2W) and high b-value DWI images to compensate for intensity non-uniformities using N4 bias correction in the SimpleITK Python v3.9 library. This was followed by basic intensity normalization, whereby voxel intensities of the entire image were scaled and shifted to a mean signal value of 300 and a standard deviation of 100. At last, an isotropic resolution of 1 × 1 × 1 mm^3^ and fixed bin-width (FBW) discretization equal to 10 were performed to handle differences in image resolution.

All the pre-processing steps were applied using the open-source software Pyradiomics v1.3.0. [21]. Radiomic features were extracted from the pre-processed T2W and DWI images using the Pyradiomics v1.3.0, in accordance with the Imaging Biomarker Standardisation Initiative (IBSI) guidelines to ensure standardized and reproducible feature computation [21,22]. In particular, the extracted texture features included (i) shape-based features (*n* = 14), (ii) first-order features (*n* = 18), (iii) gray-level co-occurrence matrix (GLCM) (*n* = 22) features, (iv) gray-level size zone matrix (GLSZM) features (*n* = 19), (v) gray-level run length matrix (GLRLM) features (*n* = 14) and vi) gray-level dependence matrix (GLDM) features (*n* = 14). These features are enabled in the Pyradiomics v1.3.0 code by default [21].

### 2.7. Statistical Analysis

Standard statistical methods were performed with SPSS software v.29.0.0 to assess longitudinal changes and evaluate correlations with radiation dose at three predefined time points [23].

Specifically, Repeated Measures Analysis of Variance (RM-ANOVA) was implemented to analyze longitudinal changes across time. Pairwise comparisons were performed between (i) baseline and mid-treatment (t0 vs. t1), (ii) mid-treatment and post-treatment (t1 vs. t2), and (iii) baseline and post-treatment (t0 vs. t2). Sphericity was examined using Mauchly’s test, and where this assumption was violated, Greenhouse–Geisser correction was applied. Post hoc pairwise comparisons between time points (t0 vs. t1, t1 vs. t2, and t0 vs. t2) were performed using Bonferroni correction. Analyses were conducted on complete cases only.

Furthermore, linear regression analysis evaluates the association between quantitative imaging parameters and Gleason Score (GS) values.

The effect of androgen deprivation therapy (ADT) schedules and radiotherapy regimens on quantitative imaging biomarkers was also assessed using RM-ANOVA, with these variables included as covariates in the statistical models where applicable.

Linear mixed models (LMMs) were implemented to investigate the relationship between radiation dose and imaging-derived parameters, accounting for repeated measurements within subjects. Radiation dose was modeled as a categorical fixed effect using indicator variables corresponding to predefined dose levels; no assumption of linearity was imposed. All models included random intercepts and slopes to account for within-subject variability, and residual diagnostics were used to verify model assumptions. LMMs accommodate missing data under the missing-at-random (MAR) assumption, whereas RM-ANOVA was restricted to complete cases.

Given the large number of extracted radiomic features, *p*-values derived from RM-ANOVA (Pillai’s Trace) were adjusted for multiple comparisons using the Benjamini–Hochberg false discovery rate (FDR) correction, with a significance threshold of q < 0.05. Pairwise comparisons were considered exploratory and are reported as uncorrected *p*-values.

No formal multiplicity correction was applied to regression or linear mixed-effects model analyses, as these were hypothesis-driven and outcome-specific.

A two-sided *p*-value of <0.05 was considered statistically significant for all non-FDR-corrected analyses.

## 3. Results

The patients’ ages ranged from 62 to 80, with a mean (M) age of 73.55 years. Baseline PSA levels ranged from 4.73 to 63.8 ng/mL, with a mean (M) value 14.97 ng/mL. Most patients were diagnosed through routine screening, while fewer presented with clinical symptoms such as urinary frequency, genital pain and dysuria. Three patients only reported a family history of prostate cancer, indicating a possible genetic predisposition. From the initial cohort, one participant declined to undergo the third MRI examination, resulting in the absence of t2 data. Furthermore, six participants were excluded from T2* mapping due to motion artifacts observed during the mFFE MRI acquisition. Further information about patient characteristics is shown in Table 2.

### 3.1. Longitudinal Changes in MRI-Derived Parameters

Mauchly’s test for Volume and T2 showed a violation of sphericity (*p* < 0.05); therefore, Greenhouse–Geisser-corrected degrees of freedom were used. In contrast, ADC and T2* satisfied the sphericity assumption (*p* > 0.05), and standard F-tests were applied.

RM-ANOVA revealed a significant effect of time on Volume (F (1.32, 26.43) = 21.20, *p* < 0.001, η^2^ = 0.52). Post hoc Bonferroni comparisons showed a significant increase from t0 (M = 44.22, standard deviation (SD) = 21.26) to t1 (M = 51.11, SD = 22.36) (*p* < 0.001), followed by significant decline from t1 to t2 (M = 37.98, SD = 15.56) (*p* < 0.001) and from t0 to t2 (*p* = 0.034).

A significant time effect was also reported for T2 values (F (1.44, 28.82) = 10.69, *p* < 0.001, η^2^ = 0.35). However, post hoc analysis revealed no significant change was found between t0 (M = 106.00, SD = 23.74) and t1 (M = 108.65, SD = 12.69), while T2 values were significantly lower at t2 (M = 93.33, SD = 9.50) compared to both t1 (*p* < 0.001) and t0 (*p* = 0.023), indicating that the overall effect was primarily driven by changes at the final time point.

For ADC, the overall effect of time was borderline significant (F (2, 40) = 3.25, *p* = 0.049, η^2^ = 0.14), but no significant pairwise differences were observed after Bonferroni correction.

No significant effect of time was observed for T2* values (F (2, 30) = 2.35, *p* = 0.132), and all post hoc comparisons were non-significant.

Mean values of parameters and detailed statistical results are summarized in Table 3.

**Figure 1:**
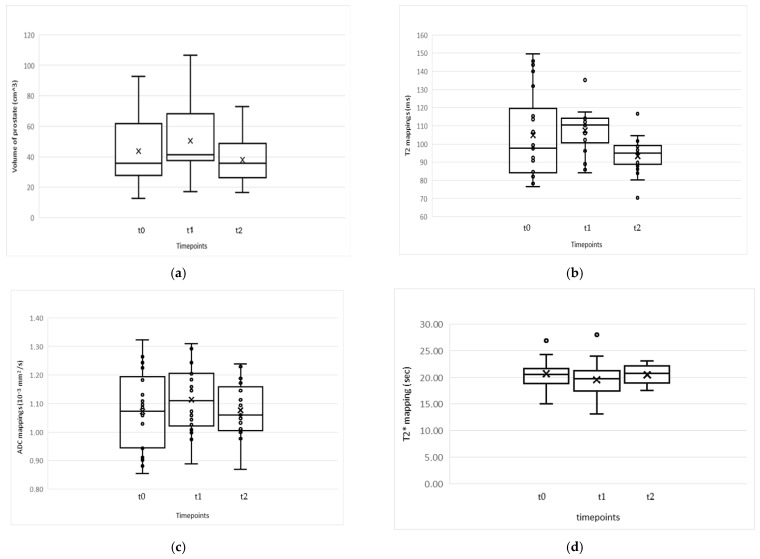
Boxplot illustration of longitudinal changes in (**a**) Volume, (**b**) T2 relaxation time, (**c**) ADC and (**d**) T2* relaxation time during radiotherapy.

Boxplots illustrate the distribution of each variable across the three time points in Figure 1.

### 3.2. Correlations Between Clinical/Treatment Factors and Prostate Imaging Parameters

The correlations between prostate imaging parameters and clinical variables were examined using linear regression analysis and the RM-ANOVA test.

Baseline values of Volume, T2 relaxation times, ADC and T2* relaxation time provided a limited correlation with GS (Volume: B = −0.013, R^2^ = 0.103, *p* = 0.145; T2: R = 0.242, R^2^ = 0.059, B = 7.697, *p* = 0.277; ADC: R = 0.214, R^2^ = 0.046, B = −0.214, *p* = 0.339; T2*: R = 0.217, R^2^ = 0.047, B = 69.585, *p* = 0.419), with regression coefficients not reaching statistical significance.

Neither ADT therapy nor radiotherapy regimen showed a significant effect on Volume, T2 relaxation times, ADC or T2* relaxation time, as shown in Table 4. Notably, the association between T2 relaxation time and hormone therapy reached statistical significance (*p* = 0.028).

Overall, Gleason Score, hormone therapy, and radiotherapy regimen exhibited limited predictive value for baseline or longitudinal changes in Volume, ADC, and T2*, although hormone therapy was associated with a parameter-specific association with T2 relaxation time.

It is worth noticing that these findings should be interpreted with caution due to potential residual confounding from treatment heterogeneity.

### 3.3. Correlation Analysis of the MRI Parameters with Radiation Dose

The effect of radiation dose on MRI-derived parameters (T2*, ADC, T2 relaxation time, and Volume) were investigated using LMMs. A consistent model structure was applied across all outcomes, with radiation dose treated as a fixed effect and subject-specific random effects included to account for within-subject repeated measurements. All models included random intercepts and random slopes for dose. The results of the statistical analysis are represented in Table 5. Each MRI-derived outcome was analyzed separately using an identical linear mixed-effects model specification.

### 3.4. Volume

**Figure 2:**
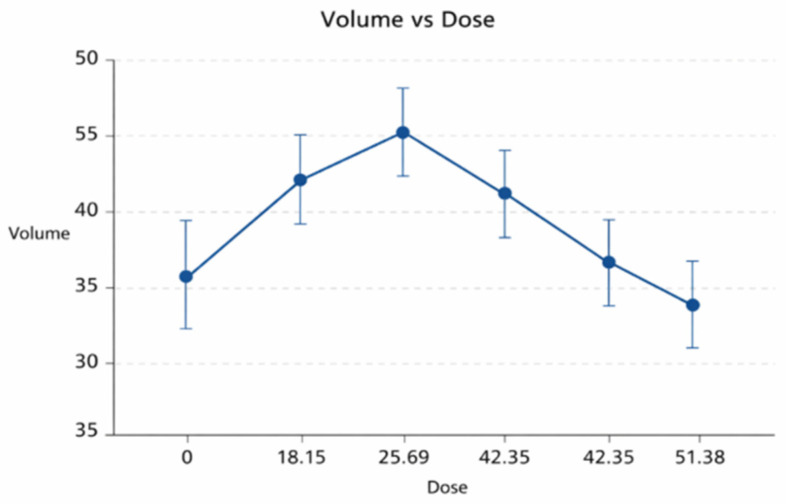
Dose–response relationship between radiation dose and volume estimated from the linear mixed-effects model.

The analysis revealed that radiation dose was significantly associated with prostate gland volume (F (4, 21.61) = 15.13, *p* < 0.001). Estimated marginal means showed an increase from 43.73 ± 4.45 cm^3^ at 0.00 μGy to 53.25 ± 4.73 cm^3^ at 25.69 μGy, followed by a decrease to 37.79 ± 5.32 cm^3^ and 36.99 ± 5.80 cm^3^ at 42.35 μGy and 51.38 μGy, respectively, suggesting a non-linear inverted U-shaped association between radiation dose and volume. This inverted U-shaped pattern reflects differences in estimated marginal means across discrete dose levels rather than a parametrically modeled nonlinear relationship. (Figure 2).

The corresponding linear mixed-effects model describing the association between volume and radiation dose is given by:

Volume_ij_ = 43.73 + 5.21⋅D_18.15_ + 9.52⋅D_25.69_ − 5.94⋅D_42.35_ − 6.74⋅D_51.38_ + u_0j_ +u_1j_⋅D_i_ + ϵ_ij_ (1)

where D_x_ denotes dose-specific dummy variables, u_0j_ represents the subject-specific random intercept, u_1j_ the random slope for dose (variance = 108.92), and $\varepsilon_{ij}$ the residual error.

### 3.5. T2 Relaxation Time

**Figure 3:**
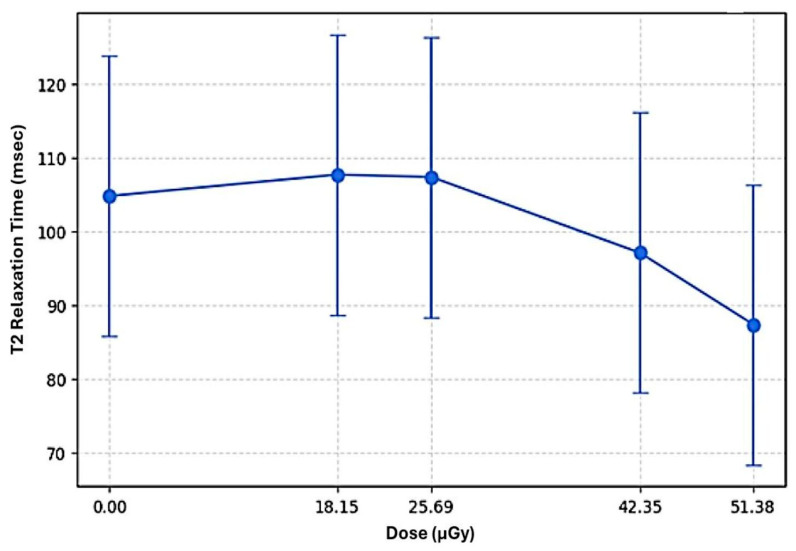
Dose–response relationship between T2 relaxation time and radiation dose estimated from the linear mixed-effects model.

Similarly, T2 relaxation time was linear correlated with the applied radiation dose (F (4, 37.57) = 6.356, *p* < 0.001). Estimated marginal means demonstrated an initial increase in T2 values from 104.75 ± 19.01 ms at 0.00 μGy to 107.32 ± 19.01 ms at 25.69 μGy, followed by a decrease to 97.03 ± 19.01 ms and 87.32 ± 19.01 ms at 42.35 μGy and 51.38 μGy, respectively. This pattern suggests a non-linear inverted U-shaped relationship between dose and T2. This inverted U-shaped pattern reflects differences in estimated marginal means across discrete dose levels rather than a parametrically modeled nonlinear relationship (Figure 3).

The association between T2 relaxation time and radiation dose was modeled using a linear mixed-effects framework, expressed as follows:

T2_ij_ = 104.75 + 2.90⋅D_18.15_ + 2.57⋅D_25.69_ − 7.72⋅D_42.35_ − 17.43⋅D_51.38_ + u_0j_ + u_1j_⋅D_i_ + ϵ_ij_ (2)

where D_x_ denotes dose-specific dummy variables, u_0j_ represents the subject-specific random intercept, u_1j_ the random slope for dose (variance = 108.92), and $\varepsilon_{ij}$ the residual error.

### 3.6. ADC

In contrast, no statistically detectable effect of the radiation on ADC values was observed (F (4, 0) = 0.822, *p* > 0.99). Bonferroni-corrected pairwise comparisons revealed no statistically significant differences between doses. This suggests that tissue diffusivity, as reflected by ADC, remained unaffected by radiation exposure in this sample.

### 3.7. T2* Relaxation Time

Also, LLM analysis indicated that radiation dose did not significantly affect T2* values (F (4, 6229.71) = 0.034, *p* = 0.998). Bonferroni-adjusted pairwise comparisons confirmed the absence of significant differences between any dose levels (all *p* > 0.99). These findings indicate that T2* was not measurably influenced by radiation dose within the studied range.

### 3.8. Radiotherapy-Induced Changes in Radiomic Features

RM-ANOVA analysis revealed that specific T2W and DWI radiomic features derived from the prostate lesions changed significantly across time points. Table 6 reports Pillai’s Trace *p*-values for the overall effect of RT phase, partial eta squared η^2^ as a measure of effect size, Greenhouse–Geisser *p*-values for sphericity violations, linear *p*-values with partial eta squared η^2^ for linearity across phases, and pairwise comparison *p*-values, with *p* < 0.05 considered statistically significant. Pairwise comparisons between time points are also reported as exploratory analyses.

To account for multiple testing across the large number of radiomic features, *p*-values derived from RM-ANOVA (Pillai’s Trace) were adjusted using the Benjamini–Hochberg false discovery rate (FDR) correction, with statistical significance defined as q < 0.05.

For T2W features, statistically significant changes were observed in Gray-Level Non-Uniformity (GLNU) and Zone Entropy. Specifically, GLNU exhibited a moderate-to-large overall effect, showing a significant overall effect across phases (Pillai’s Trace *p* = 0.038, partial η^2^ = 0.373), significant differences between time points t_0_ and t_2_ (*p* = 0.028), and a linear trend across phases (phase linear *p* = 0.009). Zone Entropy demonstrated a significant overall phase effect (*p* = 0.044, partial η^2^ = 0.360) with a significant difference between t_0_ and t_1_ (*p* = 0.033). Additionally, the feature Imc1 differed significantly between t_1_ and t_2_ (*p* = 0.036).

DWI features exhibited more extensive and robust changes. For example, Mean Absolute Deviation (*p* = 0.004, partial η^2^ = 0.533) differed significantly between t_1_ and t_2_ (*p* = 0.020), indicating a moderate-to-large effect. Autocorrelation (*p* = 0.008, partial η^2^ = 0.499) showed significant differences between t_0_ and t_1_ (*p* = 0.022), and Joint Average (*p* = 0.008, partial η^2^ = 0.498) differed between t_1_ and t_2_ (*p* = 0.010). Cluster Tendency (*p* = 0.002, partial η^2^ = 0.592), Sum Squares (*p* = 0.002, partial η^2^ = 0.591), Gray-Level Variance (*p* = 0.003, partial η^2^ = 0.572), and Gray-Level Variance (*p* = 0.003, partial η^2^ = 0.572) showed significant differences between t_1_ and t_2_ (*p* < 0.05 for all). Imc1 also differed between t_0_ and t_1_ (*p* = 0.048).

Other features, including Entropy, Interquartile Range, Maximum, Range, Robust Mean Absolute Deviation, Variance, Contrast, Difference Average, Difference Variance, Idn, High Gray-Level Run Emphasis, Long-Run High Gray-Level Emphasis, Short-Run High Gray-Level Emphasis, High Gray-Level Zone Emphasis, Small-Area Gray-Level Emphasis, High Gray-Level Emphasis, and Small-Dependence High Gray-Level Emphasis, demonstrated a significant overall effect across phases, yet pairwise comparisons were not significant, indicating subtle or non-systematic temporal variations.

After FDR adjustment (q < 0.05), the majority of DWI-derived features, particularly those related to intensity variability and texture heterogeneity, remained statistically significant, whereas only a limited number of T2W features retained significance. This supports the robustness of the observed DWI findings and suggests that radiotherapy-induced changes are primarily captured by diffusion-based radiomic descriptors rather than T2W features.

Overall, these findings indicate that radiotherapy induces measurable temporal changes in prostate lesion heterogeneity, predominantly reflected in DWI-derived radiomic features, while T2W features show more limited and less robust alterations.

### 3.9. Short-Term Treatment Response Evaluation

An effective therapeutic response was defined as a reduction in PSA levels compared to baseline and the absence of a PSA rise ≥ 2 ng/mL above the nadir at 2 months following radiotherapy (RT). Patients not meeting these criteria were classified as non-responders (Table 7).

Particularly, nineteen out of twenty-two (86.37%) were classified as responders. Only two patients (9.09%) showed a significant increase in PSA following RT and metastatic progression, corresponding to non-responding cases. Furthermore, follow-up assessments at later time points were not available for certain patients (4.54%), restricting the complete analysis of therapy response.

**Figure 4:**
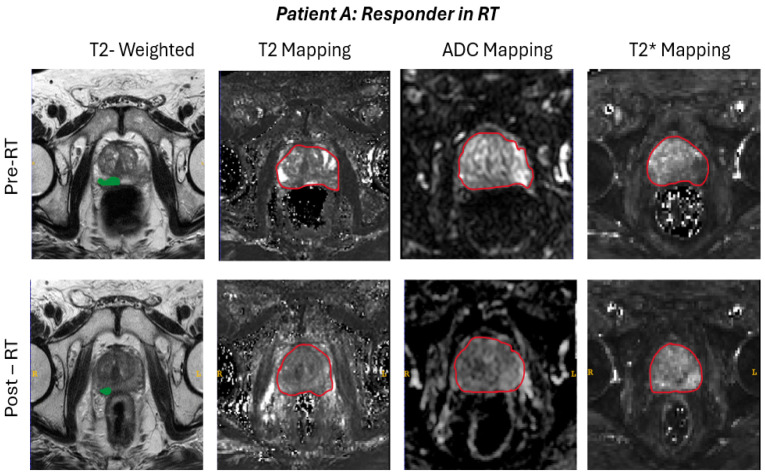

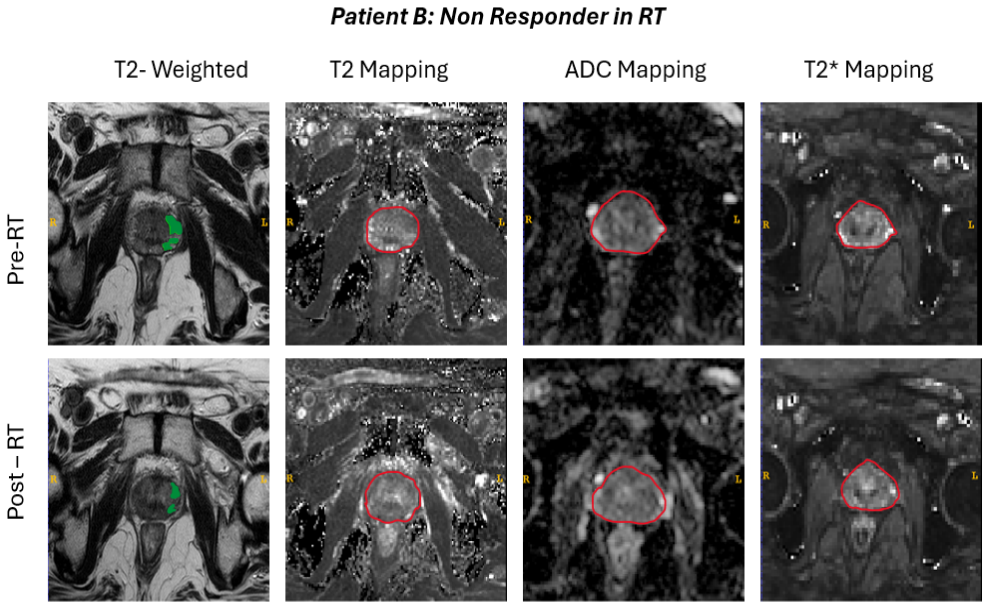
Multiparametric magnetic resonance imaging (mpMRI) of the prostate before and after radiotherapy in two patients. T2-weighted (T2w), T2 mapping, ADC mapping, and T2* mapping sequences are presented. In the upper panel (Patient A), post-RT changes consistent with therapeutic response are observed, including reduction in lesion size, increased ADC values, and signal normalization on T2w images and quantitative maps. In the lower panel (Patient B), no substantial response is evident, with persistently low ADC values, ongoing diffusion restriction, and absence of significant morphological or quantitative changes. Green markings indicate the lesions/region of interest (ROI) used for radiomics analysis, whereas red markings represent the whole-prostate segmentation used for mapping in this study. ADC: Apparent diffusion coefficient; RT: radiotherapy.

Representative examples of imaging-based short-term response assessment are illustrated in Figure 4. In the responder case (Patient A), post-RT (t2 time point) mpMRI demonstrates findings consistent with treatment effect, including reduced lesion conspicuity, increased ADC values indicating decreased diffusion restriction, and signal normalization on T2-weighted and quantitative maps. In contrast, the non-responder case (Patient B) shows persistent imaging features of active disease, with sustained diffusion restriction (low ADC), stable or minimally changed lesion morphology, and absence of significant alterations across parametric maps. These imaging patterns are in agreement with the biochemical response classification based on PSA kinetics.

## 4. Discussion

The findings of the present study are positioned in a rapidly evolving domain in which advanced quantitative MRI biomarkers and radiomics analyses are increasingly explored for monitoring radiation-induced tissue alterations to optimize PCa management. Traditional biomarkers, such as PSA, present limited reliability, especially after focal treatments, emphasizing the need for objective and precise imaging-derived metrics. Under these circumstances, analysis of the quantitative MRI imaging biomarkers and radiomics features during RT provides a comprehensive and state-of-the-art strategy that may enhance individualized monitoring, supporting the integration of advanced imaging into clinical decision-making post-RT. Equally of scientific interest is understanding the relationship between quantitative MRI parameters and the administered radiation dose, as these metrics may provide a non-invasive assessment into radiotherapy-driven microstructural and biophysical tissue changes, thereby supporting dose-adaptive and personalized PCa management. However, these findings should be interpreted in the context of early (acute and subacute) post-radiotherapy imaging changes, as the follow-up period was limited to 2 months and does not allow for assessment of long-term clinical outcomes. Therefore, the association between imaging biomarkers and long-term clinical endpoints cannot be established in the present study.

The longitudinal changes in MRI-derived parameters among radiotherapy are one significant result of this research study. Prostate volume showed a dynamic longitudinal response during RT. At mid-treatment, a notable rise of 16.5% was observed, followed by a reduction of 25.14% after treatment completion, with final values falling by 13.2% relative to baseline. This pattern may reflect radiation-induced tissue changes, such as edema and subsequent resolution; however, these interpretations remain speculative, as no histopathological validation was available. Moreover, the study highlighted that clinical variables such as GS, RT protocols, and hormone therapy seem to be independent of prostate volume. Slightly different results were reported by Gunnlaugsson et al. [24]. The authors observed a ~14% increase in prostate volume at mid-treatment and an elevation (~9%) remaining at the end of therapy [24]. The authors attributed this response to the use of extreme hypofractionated radiotherapy (7 × 6.1 Gy), which may induce more pronounced edema compared to conventional fractionation schedules [24]. The discrepancies may be explained by variations in treatment protocols, in the use of ADT, in volume calculation methods, and in the timing of MRI acquisition. Notably, MRI assessment in Gunnlaugsson et al. was conducted at last RT session, whereas in the present study imaging was performed two months after treatment completion, allowing edema to subside [24].

RM-ANOVA revealed a significant effect of time on T2 relaxation values. There was no significant difference between baseline (t0: M = 106.00, SD = 23.74) and early post-treatment (t1: M = 108.65, SD = 12.69; *p* > 0.99). At follow up, there was a considerable reduction (t2: M = 93.33, SD = 9.50; *p* < 0.001), likely due to delayed post-radiotherapy histological changes such as fibrosis. The lack of statistical significance between t0 and t2 (*p* = 0.023) underscores that early post-treatment evaluation may not reflect delayed tissue alterations. No significant effect of RT regimen or correlation with GS was observed on T2 relaxation time. However, it is noteworthy that hormone therapy had a statistically significant effect on overall T2 values (*p* = 0.028), indicating that the two groups differed in their mean T2 relaxation times. These findings indicate that T2 mapping provides temporal insights into prostate tissue changes to radiotherapy.

Quantitative T2 mapping has been recognized as a sensitive tool for prostate cancer (PCa) evaluation. Chatterjee et al. reported lower T2 values in tumor tissue (105 ± 28 ms) in contrast with normal prostate (211 ± 71 ms), with moderate negative correlation with GS (*p* = −0.284) and higher positive predictive value than conventional T2W imaging [25]. Bucher et al. demonstrated that accelerated T2 mapping distinguishes clinically significant PCa from normal tissue (tumor ~81 ms vs. normal ~152 ms; AUC = 0.973) and correlates with PI-RADS scores [26]. Mai et al. confirmed the discriminative ability of T2 (AUC = 0.871) and its negative correlation with GS (r = −0.261), with strong correlation with ADC values (r = 0.772) [27]. Overall, T2 relaxation time is a reliable imaging biomarker for the evaluation of PCa. Nonetheless, current evidence does not support its use as an independent indicator of RT response, consistent with Zacharaki et al.’s study [28]. T2 mapping therefore may offer a valuable approach for longitudinal monitoring of tissue changes, but it necessitates more validation as a quantitative biomarker for therapy efficacy.

Subsequently, analysis of the ADC maps revealed no statistically significant differences across time points. This may indicate the relative stability of the diffusion metrics at the whole-prostate level. The absence of statistically significant changes in ADC in the current study may be attributed to the analysis of the entire prostate rather than being restricted to the neoplastic lesion. The inclusion of normal prostatic tissue probably diminishes localized diffusion changes that are otherwise evident when focusing exclusively on tumor tissues, as demonstrated in the following trials. In addition, the limited sample size and associated reduced statistical power may have further contributed to the absence of statistically significant findings. As a result, our findings may indicate that post-radiotherapy changes in ADC are more easily observable at the lesion level, reflecting the greater radiosensitivity of tumor tissue relative to normal prostate [29]. This factor may also explain the lack of ADC correlation with GS, and the lack of a significant association of ADC values with ADT therapy or radiotherapy protocols.

The role of ADC as a quantitative imaging biomarker for assessing tumor response after RT in PCa has been investigated in several studies. Early investigations by Song et al. and Iannelli et al. demonstrated a consistent elevation in ADC values within malignant regions following RT, suggesting that radiation-induced cellular damage leads to reduced tumor cell density and expanded extracellular space, whereas values in normal prostate tissue remained relatively stable [30,31,32]. Longitudinal studies further substantiate these observations. For instance, Wu et al. reported a progressive increase in tumor ADC values for up to 12 months after RT delivered using VMAT [33]. Liu et al. also observed significant increases in ADC values when combining androgen deprivation therapy (ADT) with IMRT [34]. As a consequence, tumor recurrence was linked to lower post-treatment ADC values, suggesting that diffusion parameters may have prognostic values.

Despite these consistent findings, ADC variability was reported across various studies. Prostate atrophy and structural changes induced by pre-treatment androgen suppression may significantly influence baseline ADC measurements, as shown by Iraha et al. [35]. Nevertheless, more recent research suggests that ADC may also be implemented as an early biomarker of RT response. The studies by Decker et al. and Park et al. referred to a trend of increasing ADC values during the first week of treatments before biochemical changes, potentially providing earlier insight into tumor response [36,37]. Similarly, the early predictive value of ADC appears to persist even in treatment regimens when combining RT with hormone therapy. Studies by Franco et al. and McPartlin et al. shown higher ADC values after hormonotherapy in tumor tissues, supporting the inverse relationship between cellularity and diffusion [12,38].

Lastly, it is noteworthy that the integration of combined analysis of quantitative MRI parameters has gained increasing attention, contrary to diffusion imaging alone. Studies by van Schie et al. and Foltz et al. evaluated T2 and ADC changes in MRI at three or more time points during radiotherapy, aiming to detect tissue- and tumor-specific response patterns. T2 relaxation times in normal prostate tissue progressively declined during treatment, whereas malignant lesions showed minimal variation. On the other hand, ADC values increased in tumor regions while remaining relatively constant or slightly decreasing in normal tissues, indicating different microstructural responses to radiation and suggesting that weeks six to seven may be a crucial framework for detecting early imaging biomarkers [32,39]. Fernandes et al.’s study underscores the significance of mp-MRI for identifying tumor recurrence, showing persistent high T2 and low ADC in malignant tissues and enhancement vascular parameters (Ktrans, Kep) in patients with biochemical relapse [40].

These quantitative changes in T2 and ADC not only reflect tissue- and tumor-specific responses to radiation but also form the biological basis for the observed dose- and time-dependent alterations in radiomic features, highlighting the complementary value of combining conventional quantitative maps with radiomic analysis. Τhe findings of radiomic analysis revealed dose-dependent changes in both T2W and DWI features, with the most pronounced and consistent changes detected in the DWI-derived features. Previous studies have similarly reported that T2W- and DWI- based radiomics may demonstrate treatment-related changes during or after radiotherapy, demonstrating radiation-induced microstructural changes [41,42,43]. In particular, DWI-based radiomic features reflecting intensity variability and texture heterogeneity may be related to radiation-induced changes in tumor cellularity and tissue microstructure [44,45,46]. Importantly, while several features remained significant after false discovery rate (FDR) correction, radiomic features should be considered exploratory biomarkers rather than validated clinical endpoints. Their biological meaning requires further validation through correlation with histopathology and clinical outcomes.

Nevertheless, although quantitative MRI and radiomics approaches show considerable promise for characterizing radiation-induced tissue changes, their integration into routine radiotherapy assessment remains under active investigation. These observed dose–temporal variations offer a basis for the construction of machine learning models for evaluating RT response [45,47,48,49,50]; however, examining this dynamic was beyond the scope of the present study.

T2* relaxation times remained stable across all time points (t0, t1, t2; *p* > 0.13), and no statistically significant differences were detected in any pairwise comparisons. This apparent stability is likely attributable to a combination of inherent measurement variability and the relatively low sensitivity of T2* to subtle treatment-induced microstructural alterations in this cohort. Therefore, the observed variability likely captures an inherent data variability rather than treatment-related changes. In contrast, Wang et al.’s study showed significantly decreased post-radiotherapy R2* along with notable changes in other parameters (ADC, Kep, Ktrans), reflecting treatment response and indicating the potential utility for monitoring tumor oxygenation and microenvironment [51].

Most studies have highlighted T2* mapping as a non-invasive imaging metric for differentiating malignant and benign prostate lesions and assessing tumor grade. Specifically, Zhou et al. implemented oxygen-enhanced (OE) MRI into a multiparametric protocol, showing enhanced R2* in tumor regions, associated with ADC and GS [52]. Kim et al. and Wu et al. similarly noted elevated R2 * or reduced T2* in tumor areas, with Wu demonstrating improved diagnostic capability with ADC [53,54]. More recently, Wenhao et al. linked T2* with International Society of Urological Pathology (ISUP) grade and tissue iron, achieving high discrimination of PCa from benign prostatic hyperplasia (BPH) (AUC ≈ 0.865–0.867) [55]. Collectively, T2* mapping appears as a robust non-invasive tool for PCa diagnosis, evaluation of tumor aggressiveness, and post-therapy follow-up, offering valuable information on tissue hypoxia and therapeutic response.

At the end of this study, radiation dose showed a statistically significant association with changes in prostate volume and T2 relaxation time (*p* < 0.001). These findings suggest that RT-induced tissue changes, such as edema, inflammatory responses, and increased free water content, may be detectable through MRI-derived parameters. In contrast, ADC (*p* > 0.99) and T2* values (*p* = 0.998) are consistent with the biological specificity of these parameters. ADC reflects microstructural water diffusivity and cellularity, and T2* is influenced by magnetic susceptibility effects and hemodynamic factors, which may remain relatively stable after radiation-induced tissue changes. Nevertheless, despite promising findings from experimental and clinical studies, the quantitative relationship between MR-derived parameters and the actual absorbed radiation dose remains limited [56,57]. Additionally, since radiation dose was intrinsically linked to treatment progression and acquisition time points (baseline, mid-treatment, and post-treatment), the observed effects likely reflect a combination of dose- and time-related changes rather than an isolated dose–response relationship. Therefore, dose and temporal effects cannot be fully disentangled within the present study design, which should be acknowledged as a methodological limitation.

Although short-term PSA changes were evaluated, the present study design does not allow for robust assessment of long-term treatment response or oncological outcomes. Therefore, the observed imaging and radiomic changes should be interpreted as early correlates of radiation-induced tissue effects rather than indicators of clinical efficacy.

This study presents key strengths, including the multi-parameter assessment of four MRI imaging biomarkers (T2, ADC, T2W, T2*) and radiomics features with standardized MRI acquisition protocol among RT. Moreover, the study provides a comprehensive evaluation regarding the influence of hormone therapy, radiotherapy regimen, and administered dose on these imaging parameters, enhancing the clinical significance of the findings.

However, several limitations should be acknowledged. The relatively small sample size limits statistical power and the robustness of subgroup analyses. In addition, the restricted follow-up period does not allow for assessment of long-term clinical outcomes such as biochemical recurrence or progression-free survival. Therefore, the observed imaging changes should be interpreted as early (acute and subacute) treatment-related effects. From a methodological perspective, the direct comparability between these two approaches may be limited, as T2, ADC, and T2* analyses were based on whole-organ segmentation, whereas radiomic features were derived from focal tumor segmentation. Whole-organ segmentation was necessary due to small and low GS lesions that were difficult to detect in the respective sequences in 10 out of 22 patients. Consequently, the assessment of dose effects was performed across the entire prostate gland rather than on individual lesions. In addition, although treatment variables (hormone therapy and radiotherapy regimen) were included in the statistical models as covariates where appropriate, residual confounding cannot be excluded due to the limited sample size.

Future work should focus on larger prospective cohorts with extended follow-up to evaluate prognostic performance. Further validation of radiomic features against histopathology and clinical endpoints, including PSA dynamics, is also required. In addition, standardized image registration and comprehensive reproducibility assessments—including inter- and intra-observer variability, Dice similarity coefficients across sequences, and formal test–retest analyses of radiomic feature stability—are essential to improve robustness and facilitate clinical translation.

## 5. Conclusions

This study demonstrates the potential role of quantitative MRI-derived parameters and radiomic features in characterizing PCa tissue changes during and after RT. Volume and T2 relaxation time show time alterations during and after RT, as well as a significant correlation with derived radiation dose, indicative of temporary edema and inflammatory response. ADC and T2*, by contrast, remained largely stable, suggesting limited sensitivity to whole-prostate microstructural or hemodynamic modifications at the assessed time points. Radiomic analysis, particularly from DWI, revealed minor dose- and time-dependent variations in tissue heterogeneity and microarchitecture. Overall, these findings support the use of combined MRI biomarkers and radiomic features that may provide exploratory imaging signals for assessing RT-induced tissue changes in PCa, even before clinical indicators. However, given the limited PSA data and follow-up, no conclusions can be drawn regarding clinical performance or prediction of biochemical response, and further validation in larger cohorts with adequate outcomes is required.

## Figures and Tables

**Table 1:** Treatment parameters for ultra-hypoAR and hypoAR prostate radiotherapy regimens.

Regimen	Target Volumes	Total Dose (Gy)	Fractions	Schedule	Daily Dose (Gy)	Boost Dose Per Fraction (Gy)
Ultra-hypoAR	Prostate, seminal vesicles	Prostate: 42.35 SV *: 38.5	7	2 days/week × 4 weeks	Prostate: 6.05 SV *: 5.5	Prostate: 0.25
HypoAR	Prostate, seminal vesicles, pelvic LNs *	Prostate: 51.38 SV: 49 Pelvic LNs *: 37.8	14	5 days/week × 3 weeks	Prostate: 3.67 SV *: 3.5 Pelvic LNs *: 2.7	Prostate: 0.97 SV: 0.8

* Abbreviations: SV = seminal vesicles, LNs = lymph nodes.

**Table 2:** Clinical and epidemiological characteristics of the patient cohort.

Variable	Value
Patients	22
Histopathologically confirmed malignant lesions	22
Mean age (years) (mean ± SD)	73.55 ± 4.86
Mean PSA * level (ng/mL) (mean ± SD)	14.97 ± 14.82
Gleason Score (GS)	
GS * = 6 (3 + 3)	10
GS * = 7 (3 + 4)	5
GS * = 7 (4 + 3)	3
GS * = 8 (4 + 4)	3
GS * = 9 (5 + 4)	1

* SD: standard deviation; PSA: prostate-specific antigen; GS: Gleason Score.

**Table 3:** Mean values (mean ± SD) and repeated-measures ANOVA results for Volume, T2, ADC, and T2 at three time points (t0, t1, t2) during radiotherapy, including post hoc Bonferroni comparisons.

Parameter	t0 (Mean ± SD)	t1 (Mean ± SD)	t2 (Mean ± SD)	Sphericity	ANOVA RM F (df), *p*, Partial η^2^	Post Hoc Comparisons (Bonferroni)
Volume (cm^3^)	44.22 ± 21.26	51.11 ± 22.36	37.98 ± 15.56	χ^2^ (2) = 13.68, *p* = 0.001	F (1.322, 26.434) = 21.20, *p* < 0.001, η^2^ = 0.515	t0→t1: increase 6.89, *p* < 0.001; t1→t2: decrease 13.13, *p* < 0.001; t0→t2: decrease 6.24, *p* = 0.034
T2 (ms)	106.00 ± 23.74	108.65 ± 12.69	93.33 ± 9.50	χ^2^ (2) = 9.335, *p* = 0.009	F (1.441, 28.815) = 10.69, *p* < 0.001, η^2^ = 0.348.	t0→t1: NS; t1→t2: decrease15.31, *p* < 0.001; t2→t0: decrease 12.67, *p* = 0.023
ADC (×10^−3^ m^2^/s)	1.07 ± 0.028	1.12 ± 0.024	1.07 ± 0.020	χ^2^ (2) = 0.520, *p* = 0.769	F (2,40) = 3.246, *p* = 0.049, η^2^ = 0.140	t0→t1: NS, *p* = 0.106; t1→t2: NS, *p* = 0.132; t2→t0: NS, *p* > 0.99
T2* (ms)	20.7 ± 2.8	19.6 ± 3.4	20.6 ± 1.8	χ^2^ (2) 0.722 *p* = 0.102	F (2,30) = 2.35, *p* = 0.132, η^2^ = 0.25	t0→t1: NS, *p* = 0.130; t1→t2: NS, *p* > 0.99; t2→t0: NS, *p* = 0.651

Abbreviations: RM ANOVA = repeated-measures ANOVA; SD = standard deviation; NS = not significant, η^2^ = partial effect size; ADC = apparent diffusion coefficient.

**Table 4:** Effects of Gleason Score, hormone therapy, and radiotherapy regimen on prostate imaging parameters.

Parameter	Predictor	Statistic/Model	Significance (*p*)
Volume (cm^3^)	Gleason Score	B = −0.013, R^2^ = 0.103	0.145
	Hormone therapy	F = 0.264, partial η^2^ = 0.014	0.613
	Radiotherapy regimen	F = 0.023, partial η^2^ = 0.001	0.880
T2 (ms)	Gleason Score	B = 7.697, R^2^ = 0.059	0.277
	Hormone therapy	F = 5.623, partial η^2^ = 0.228	0.028
	Radiotherapy regimen	F = 1.235, partial η^2^ = 0.043	0.393
ADC (×10^−3^ mm^2^/s)	Gleason Score	B = −0.214, R^2^ = 0.046	0.339
	Hormone therapy	F = 2.776, partial η^2^ = 0.112	0.112
	Radiotherapy regimen	F = 2.78, partial η^2^ = 0.127	0.112
T2* (ms)	Gleason Score	B = 69.585, R^2^ = 0.047	0.419
	Hormone therapy	F = 0.780, partial η^2^ = 0.053	0.392
	Radiotherapy regimen	F = 0.149, partial η^2^ = 0.011	0.705

**Table 5:** Dose-dependent changes in T2, Volume, ADC, and T2*: linear mixed model outcomes and association patterns.

Parameter	F (df)	*p*-Value	Significant Dose Effect	Estimated Margins Mean
T2	F (4, 37.57) = 6.356	<0.001	Yes	Non-linear (inverted U-shape)
Volume	F (4, 21.61) = 15.13	<0.001	Yes	Non-linear (inverted U-shape)
ADC	F (4, 0) = 0.822	>0.99	No	No observable trend
T2*	F (4, 6229.71) = 0.034	0.998	No	No observable trend

**Table 6:** Statistically significant variables identified through repeated measures analysis.

Feature	Pillai’s Trace *p*-Value	Partial η^2^	*p*-Value Greenhouse-Geisser	Phase-Linear *p*-Value	Phase–Linear Partial η^2^	Pairwise Comparisons *p*-Value	Benjamini–Hochberg False Discovery Rate (FDR) Correction
**T2W**							
Imc1	0.46	0.355	0.069	0.135	0.142	t1 vs. t2: 0.036	0.46
Gray-Level Non -Uniformity	0.038	0.373	0.124	0.009	0.372	t0 vs. t2 0.028	0.042
Zone Entropy	0.044	0.360	0.133	0.367	0.055	t0 vs. t1: 0.033	0.047
**DWI**							
Entropy	0.046	0.355	0.110	0.059	0.218	ns	0.048
Interquartile Range	0.009	0.490	0.114	0.040	0.253	ns	0.020
Maximum	0.021	0.423	0.071	0.022	0.303	ns	0.031
Mean Absolute Deviation	0.004	0.533	0.063	0.025	0.292	t1 vs. t2: 0.020	0.019
Range	0.010	0.482	0.076	0.015	0.336	ns	0.021
Robust Mean Absolute Deviation	0.006	0.515	0.099	0.040	0.253	ns	0.020
Variance	0.002	0.589	0.069	0.027	0.287	ns	0.017
Autocorrelation	0.008	0.499	0.050	0.007	0.390	t0 vs. t1: 0.022	0.020
Joint Average	0.008	0.498	0.045	0.003	0.449	t0 vs. t2: 0.010	0.020
Cluster Tendency	0.002	0.592	0.089	0.037	0.258	t1 vs. t2: 0.012	0.017
Contrast	0.006	0.517	0.078	0.017	0.325	ns	0.020
Difference Average	0.014	0.458	0.110	0.021	0.306	ns	0.025
Difference Variance	0.020	0.429	0.101	0.029	0.278	ns	0.031
Imc1	0.011	0.476	0.043	0.016	0.329	t0 vs. t1: 0.048	0.021
Idn	0.035	0.380	0.384	0.254	0.086	ns	0.042
Sum Squares	0.002	0.591	0.086	0.035	0.263	t1 vs. t2: 0.013	0.017
Gray-Level Variance	0.003	0.572	0.171	0.102	0.168	t1 vs. t2: 0.013	0.017
High Gray-Level Run Emphasis	0.032	0.387	0.173	0.039	0.254	ns	0.042
Long-Run High Gray-Level Emphasis	0.037	0.376	0.153	0.034	0.266	ns	0.042
Short-Run High Gray-Level Emphasis	0.032	0.388	0.162	0.41	0.249	ns	0.042
Gray-Level Variance	0.003	0.572	0.171	0.102	0.168	t1 vs. t2: 0.013	0.017
High Gray-Level Zone Emphasis	0.011	0.472	0.147	0.037	0.259	ns *	0.021
Small-Area Gray-Level Emphasis	0.007	0.509	0.148	0.042	0.248	ns *	0.020
High Gray-Level Emphasis	0.034	0.382	0.176	0.039	0.254	ns *	0.042
Small-Dependence High Gray-Level Emphasis	0.017	0.441	0.195	0.065	0.209	ns *	0.028

* ns = not statistically significant.

**Table 7:** Evolution of PSA levels before and after radiotherapy in participants.

No	PSA * Before RT * (ng/mL)	PSA * 2 Months After RT * (ng/mL)	PSA * 3 Months After RT * (ng/mL)	Treatment Response
1	4.2	0.42	Ν/A	Responder
2	12.8	0.01	0.002	Responder
3	11.7	0.633	Ν/A *	Responder
4	11.6	0.166	0.052	Responder
5	11	14.17	8.2	Non-responder (distant metastasis)
6	29	0.2	0	Responder
7	22	0.02	0.02	Responder
8	5.5	2.23	0.97	Responder
9	63.8	0.06	10.01	Non-responder
10	38	0.07	Ν/A *	Responder
11	7	3.8	2	Responder
12	12.8	3.17	1.14	Responder
13	45	10.07	0.07	Responder
14	8.9	Ν/A *	Ν/A *	Ν/A
15	4.95	1.56	1.38	Responder
16	7.64	2.2	Ν/A *	Responder
17	4.73	0.02	0.006	Responder
18	7.4	1.4	Ν/A *	Responder
19	8	0.03	0.27	Responder
20	10	2.24	Ν/A *	Responder
21	7.4	1.44	Ν/A *	Responder
22	7.11	0.02	Ν/A *	Responder

* Abbreviations: PSA: prostate-specific antigen; RT: radiotherapy; N/A: not available.

## Data Availability

Data is unavailable due to privacy or ethical restrictions.

## Abbreviations

The following abbreviations are used in this manuscript:

PSA	Prostate-Specific Antigen
MRI	Magnetic Resonance Imaging
PCa	Prostate Cancer
ADC	Apparent Diffusion Coefficient
AΝOVA	Analysis of Variance
mpMRI	Multiparametric Magnetic Resonance Imaging
RT	Radiotherapy
NICE	Health and Care Excellence
ESUR	European Society of Urogenital Radiology
ESUI	European Society of Urogenital Imaging
PI-RADS	Prostate Imaging Reporting and Data System
PI-RR	Prostate Imaging for Recurrence Reporting
bpMRI	Biparametric Magnetic Resonance Imaging
DWI	Diffusion-Weighted Imaging
mFFE	Multi-Echo Fast Field Echo
TSE	Turbo Spin Echo
TE	Time Echo
SIB-VMAT	Simultaneous Integrated Volumetric Modulated Arc Therapy
IGRT	Image-Guided Radiotherapy
TRS	Treatment Planning System
ultra-hypoAR	Ultra-Hypofractionated Accelerated Regimen
hypoAR	Hypofractionated Accelerated Regimen
ICRU	International Commission on Radiation Units & Measurements
PTV	Planning Treatment Volume
CBCT	Cone-Beam Computed Tomography
ADT	Androgen Deprivation Therapy
LH-RH	Luteinizing Hormone–Releasing Hormone
T2W	T2-Weighted
FBW	Fixed Bin Width
IBSI	Imaging Biomarkers Standardization Initiative
GLCM	Gray-Level Co-Occurrence Matrix
GLSZM	Gray-Level Size Zone Matrix
GLRLM	Gray-Level Run Length Matrix
GLDM	Gray-Level Dependence Matrix
RM-ANOVA	Repeated Measures Analysis of Variance
GS	Gleason Score
LMM	Linear Mixed Model
M	Mean
SD	Standard Deviation
GLNU	Gray-Level Non-Uniformity
post-RT	Post-Radiotherapy
OE	Oxygen-Enhanced MRI
ISUP	International Society of Urological Pathology
BPH	Benign Prostatic Hyperplasia
